# *KDF1* Novel Variant Causes Unique Dental and Oral Epithelial Defects

**DOI:** 10.3390/ijms232012465

**Published:** 2022-10-18

**Authors:** Miao Yu, Hangbo Liu, Yang Liu, Jinglei Zheng, Junyi Wu, Kai Sun, Hailan Feng, Haochen Liu, Dong Han

**Affiliations:** Department of Prosthodontics, Peking University School and Hospital of Stomatology & National Center of Stomatology & National Clinical Research Center for Oral Diseases & National Engineering Research Center of Oral Biomaterials and Digital Medical Devices, 22 Zhongguancun South Avenue, Haidian District, Beijing 100081, China

**Keywords:** *KDF1*, tooth agenesis, dental abnormality, gingival epithelium abnormality

## Abstract

*Keratinocyte differentiation factor 1* (*KDF1*) is a recently identified and rare candidate gene for human tooth agenesis; however, *KDF1*-related morphological characteristics and pathological changes in dental tissue and the oral epithelium remain largely unknown. Here, we employed whole-exome sequencing (WES) and Sanger sequencing to screen for the suspected variants in a cohort of 151 tooth agenesis patients, and we segregated a novel *KDF1* heterozygous missense variation, c.920G>C (p.R307P), in a non-syndromic tooth agenesis family. Essential bioinformatics analyses and tertiary structural predictions were performed to analyze the structural changes and functional impacts of the novel KDF1 variant. The subsequent functional assessment using a TOP-flash/FOP-flash luciferase reporter system demonstrated that *KDF1* variants suppressed the activation of canonical Wnt signaling in 293T cells. To comprehensively investigate the *KDF1*-related oral morphological anomalies, we performed scanning electron microscopy and ground section of the lower right lateral deciduous incisor extracted from #285 proband, and histopathological assessment of the gingiva. The phenotypic analyses revealed a series of tooth morphological anomalies related to the *KDF1* variant R307P, including a shovel-shaped lingual surface of incisors and *cornicione*-shaped marginal ridges with anomalous morphological occlusal grooves of premolars and molars. Notably, keratinized gingival epithelium abnormalities were revealed in the proband and characterized by epithelial dyskeratosis with residual nuclei, indistinct stratum granulosum, epithelial hyperproliferation, and impaired epithelial differentiation. Our findings revealed new developmental anomalies in the tooth and gingival epithelium of a non-syndromic tooth agenesis individual with a novel pathogenic *KDF1* variant, broadening the phenotypic spectrum of *KDF1*-related disorders and providing new evidence for the crucial role of *KDF1* in regulating human dental and oral epithelial development.

## 1. Introduction

Tooth agenesis is a developmental and genetic disease that manifests as a reduced number of teeth, and it can be accompanied by other systemic dysplasias, or occur independently [1,2,3]. Most affected individuals suffer from variable morphological abnormalities in their remaining teeth and other craniofacial organs, which consequently cause masticatory and speech dysfunctions, or even psychological problems [4]. Environmental disturbances and genetic factors are both involved in the pathogenesis of tooth agenesis, with the latter playing a predominant role [5,6]. Over the past two decades, continuous scientific efforts have led to the decoding of a cluster of genes that encode key proteins of major developmental pathways that are currently accepted to be the etiologies for 90% of tooth agenesis; these genes include *Paired box 9* (*PAX9*), *Msh homeobox 1* (*MSX1*), *Wnt family member 10A* (*WNT10A*), *ectodysplasin A* (*EDA*), *Wnt family member 10 B* (*WNT10B*), *LDL receptor related protein 6* (*LRP6*), and *Axin 2* (*AXIN2*) [7,8,9,10,11,12,13,14,15,16,17,18,19]. However, rarely identified or unknown pathogenetic genes are observed in approximately 10% of patients with tooth agenesis, and such cases require further investigation [20].

*Keratinocyte differentiation factor 1* (*KDF1*; OMIM *616758) is the most recently identified, rare candidate gene for tooth agenesis. In 2013, mutagenesis screening of ENU-induced mutant mice (*shd*^−/−^) led to the mapping of the human gene *KDF1* to 1p36.11, which shares high homology with the mouse *Kdf1* gene [21]. A heterozygous variation in *KDF1* was first reported to cause autosomal dominant syndromic tooth agenesis, namely, ectodermal dysplasia-12 (ECTD12; OMIM #617337), which manifests as congenital absent teeth, dystrophic toenails, hypohidrosis, and hair abnormalities [22]. Subsequently, other researchers segregated a novel pathogenic *KDF1* variation into a non-syndromic tooth agenesis family [23]. This previous genetic evidence revealed the relevance of *KDF1* variations to tooth agenesis and suggested an indispensable pathogenic role of *KDF1* in tooth agenesis.

Mouse *Kdf1* signals are specifically expressed in epidermal cells through epidermogenesis and the tooth epithelium during development [21,23], and they were further proven to participate in regulating the development of skin, craniofacial organs, and limb buds [24]. *Kdf1* mutant *shd*^−/−^ fetuses present perinatal lethality, and develop defects in multiple ectodermal-derived organs, such as tight and hyperplastic skin with a damaged epidermal barrier, lack of a mouth opening, an underdeveloped digestive tract, shortened and fused limbs, a cleft palate, and a shortened snout. Molecular mechanism studies preliminarily revealed that Kdf1 balances the homeostasis of epidermal progenitor cell proliferation-differentiation by inhibiting p63 and deubiquitinating IKKα [21,24]. However, the underlying effects of *KDF1* dysfunction on the epithelial-mesenchymal interactions during tooth morphogenesis, enamel formation, and oral epithelium development have not been clarified.

In this study, we identified a previously undescribed *KDF1* heterozygous missense variation in one non-syndromic tooth agenesis family using whole-exome sequencing (WES) and Sanger sequencing. We then used bioinformatic analyses and in vitro functional assessments to predict the pathogenicity of the identified variant. In addition, we performed scanning electron microscopy, tooth ground section, and histopathological assessment to assess the enamel structure and the gingival epithelial structure. Our study elaborated on the tooth abnormalities that occur with *KDF1*-related tooth agenesis and identified, for the first time, oral epithelium defects associated with *KDF1*-related tooth agenesis.

## 2. Results

### 2.1. The Novel Heterozygous Missense KDF1 Variant Was Identified in a Tooth Agenesis Family

Variant analysis of 151 probands with tooth agenesis revealed a proband from family #285 carrying a novel heterozygous variation (NM_152365.3:c.920G>C; NP_689578.2:p.R307P) of *KDF1*. This variation was not observed in the 100 healthy controls. In our study, the detection rate of the *KDF1* variant was 0.66% in patients with tooth agenesis.

**Figure 1:**
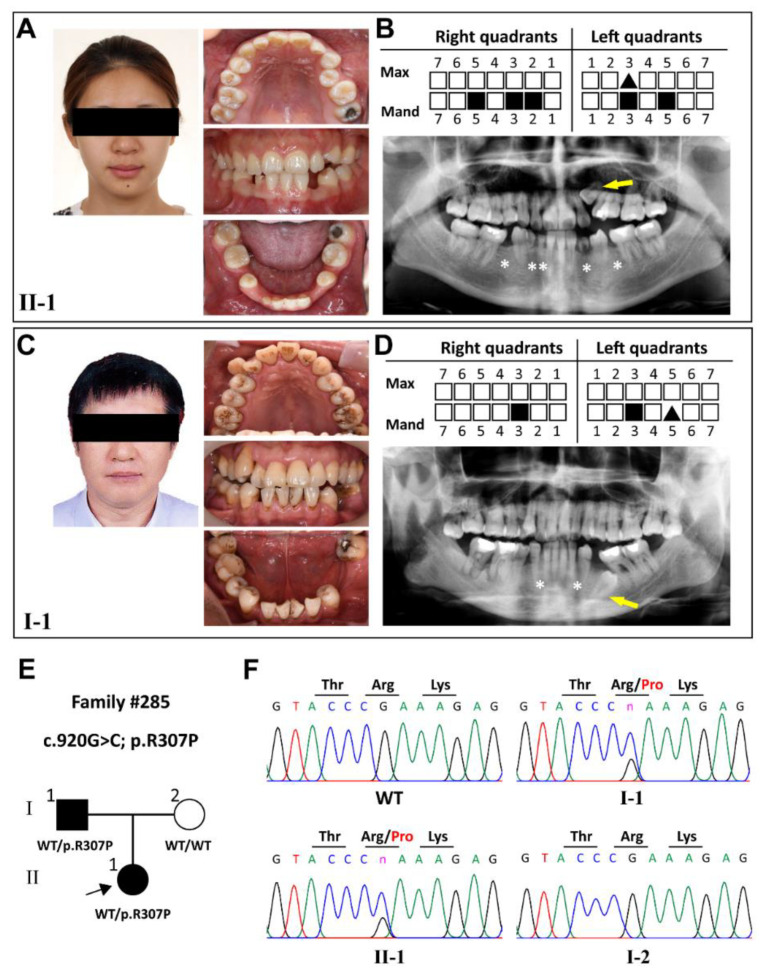
Clinical characteristics and variational analysis of patients with tooth agenesis from family #285. (**A**,**C**) Front views and intraoral photographs of #285 proband (II-1) and her father (I-1). (**B**) Schematic and panoramic radiograph of #285 II-1 showing five permanent teeth missing. (**D**) Schematic and panoramic radiograph of #285 I-1 showing two permanent teeth missing. (**E**,**F**) Pedigree and corresponding DNA sequencing chromatograms presenting a novel *KDF1* heterozygous missense variation (NM_152365.3, c.920G>C;p.R307P) identified as the genetic cause of the tooth agenesis in #285 proband (II-1), her father (I-1), and her mother (I-2). Black squares in the schematics and white asterisks in the panoramic radiographs indicate the position of a missing tooth. Black triangles in the schematics and arrows in the panoramic radiographs indicate the impacted teeth. Four bases are represented by four different colors in DNA sequencing chromatograms and the color of green indicates adenine; red indicates thymine; black indicates guanine and blue indicates cytosine. Max, maxillary; Mand, mandibular.

In family #285, clinical and radiographic examinations of proband II-1, a 21-year-old woman, revealed the congenital absence of five permanent teeth in the mandibular dentition, retention of three deciduous teeth, and one impacted maxillary canine (Figure 1A,B). Her father (I-1), 49 years old, showed congenital agenesis of two mandibular canines symmetrically and one impacted mandibular premolar (Figure 1C,D). Facial features, hair, skin, and hands were normal. No clinical symptoms were observed in her mother. Using WES and Sanger sequencing, we identified a novel *KDF1* heterozygous variation, c.920G>C;p.R307P, in proband II-1 and her father I-1 (Figure 1E,F). Our findings indicated that *KDF1* variation c.920G>C;p.R307P was inherited in an autosomal-dominant manner in this non-syndromic tooth agenesis family.

The *KDF1* variation c.920G>C;p.R307P was not listed in the dbSNP, gnomAD, or 1000 Genomes Browser database (Table 1). In silico bioinformatics tools, such as MutationTaster, PROVEAN, and SIFT, predicted that this variant would cause disease, have deleterious effects (with a score of −6.14), and produce damage (with a score of 0.000), which indicated that the variant might damage the normal physiological function of the KDF1 protein (Table 1). On the basis of current evidence of pathogenicity, the American College of Medical Genetics and Genomics (ACMG) guidelines interpret the variant as likely pathogenic (Table 1), suggesting that further molecular genetic investigations are necessary.

### 2.2. The KDF1 Novel Variation R307P Impaired the Protein Structure

**Figure 2:**
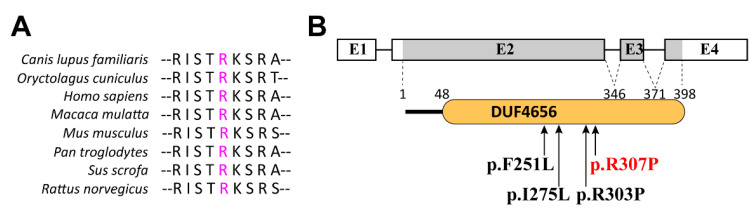

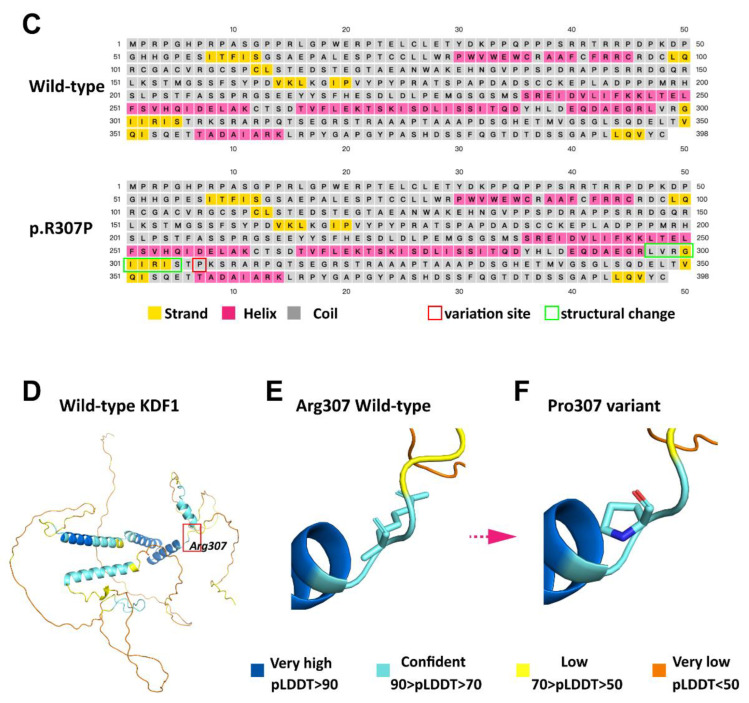
Conservation analysis, location, and predicted structural consequences of R307P and three previously reported variations in the DUF4656 domain of KDF1. (**A**) Analysis of sequence conservation of the affected amino acid in the KDF1 protein among multispecies. (**B**) Schematic diagram of the human *KDF1* gene and DUF4656 domain of the KDF1 protein showing all the KDF1 variations reported in patients with tooth agenesis are distributed in the DUF4656 domain. The novel variation identified in this study is in red, and the previously reported variations are in black. (**C**) Secondary structure analysis of the wild-type KDF1 and R307P variant. The solid squares, representing helix structures, strands, and coils, are drawn in pink, yellow, and grey, respectively. The variation site is boxed in red, and the structural changes of the affected amino acids are boxed in green. (**D**) Tertiary structure of the wild-type KDF1 protein predicted by AlphaFold. (**E**,**F**) Predicted conformational consequences of KDF1:R307P.

The variant locus arginine 307 is highly conserved across multiple species (Figure 2A) and is located in the conserved domain of unknown 4656 (DUF4656) of the KDF1 protein (Figure 2B). We conducted secondary and tertiary protein structural predictions to analyze the structural changes and functional impacts of the novel KDF1 variant. In the secondary structure, the R307P variant led to a substitution of Arg with Pro and resulted in a slight conversion of the helix-coil-strand configuration to coil-strand-coil from the 297th to 305th amino acid (Figure 2C). On the basis of the amino acid sequence and the secondary structure, the tertiary protein structure of wild-type KDF1 was predicted by the AlphaFold algorithm as a reference, and the average model confidence score (pLDDT) was 0.59 (Figure 2D). Conformational structural analysis revealed that Arg307 protrudes from the coil and the R307P variant causes arginine, a positively charged polar amino acid with a long side chain (Figure 2E), to be substituted by a nonpolar hydrophobic proline with a heterocyclic ring (Figure 2F). These structural impairments suggested that the R307P variant might severely affect the biological function of KDF1.

### 2.3. Patients Harboring the KDF1 Variant Showed Distinct Tooth Morphological Anomalies

**Figure 3:**
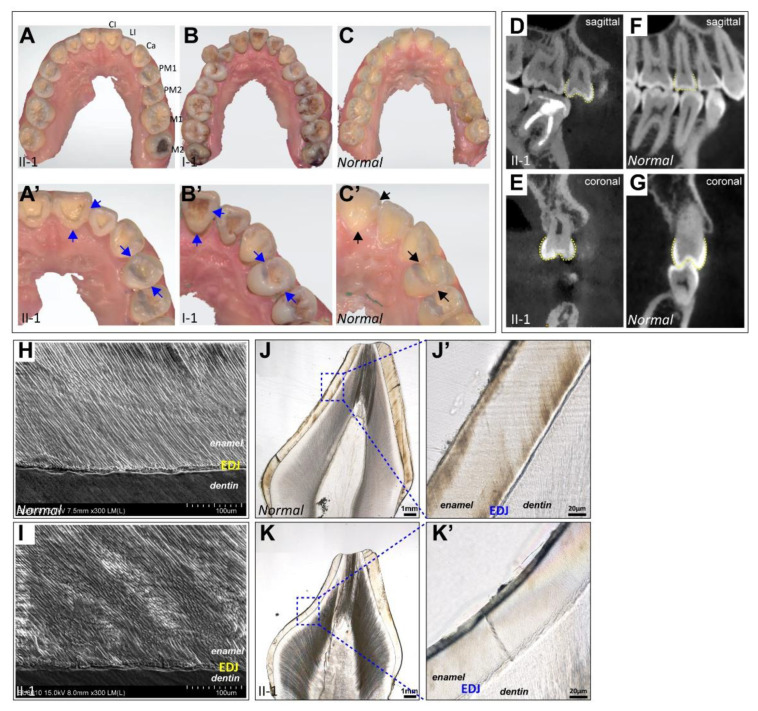
Evaluation of the permanent tooth morphology and extracted deciduous tooth structure. (**A**–**C**) Digital intraoral scanning images of the maxillary dentition of #285 proband II-1 (**A**), her father I-1 (**B**), and the normal control (**C**). (**A’**–**C’**) Detailed views of scanning images showing shovel-shaped incisors and *cornicione*-shaped premolars and molars in patients with the *KDF1* variant (indicated by the blue arrows). (**D**–**G**) CBCT images of #285 proband II-1 (**D**,**E**) and the normal control (**F**,**G**) in sagittal view showing protuberant mesial and distal marginal ridges and deep grooves of premolars in patients with the *KDF1* variant (**D**), and images in coronal view showing rounded buccal and lingual cusps and flat grooves of premolars in patients with the *KDF1* variant (**E**). (**H**,**I**) SEM images of the extracted deciduous tooth from #285 proband II-1 (**I**) and the normal control (**H**). (**J**,**K**,**J’**,**K’**) Tooth ground section images and detailed views of the enamel structure of a deciduous tooth from #285 proband II-1 and the normal control. CI, central incisor; LI, lateral incisor; Ca, canine; PM, premolar; M, molar; and EDJ, enamel-dentine junction.

After a comprehensive evaluation of the permanent tooth morphology by digital intraoral scanning and cone beam computed tomography (CBCT), we observed typical shovel-shaped incisors, which were characterized by thickened mesial-distal marginal ridges, prominent cingulum, and deep lingual fossa on the upper dentitions of the proband and her father (Figure 3A–C). When compared with the normal control, the upper premolars and molars of the two patients also manifested as apparently protuberant mesial and distal marginal ridges (Figure 3D,F), rounded buccal and lingual cusps (Figure 3E,G), and anomalous morphological occlusal grooves caused by triangular ridge defects (Figure 3D–G), thus representing *cornicione*-shaped premolars and molars (Figure 3A–C). Scanning electron microscopy (SEM) and tooth ground section results showed no obvious abnormalities in the enamel structure of the proband’s extracted deciduous teeth (Figure 3H–K); however, the effects of the *KDF1* variant on the permanent tooth structure remain unclear. These results suggest that the *KDF1* variant is associated with tooth morphogenesis.

### 2.4. KDF1 Variation R307P Resulted in Proliferation and Differentiation Defects in the Gingival Epithelium

**Figure 4:**
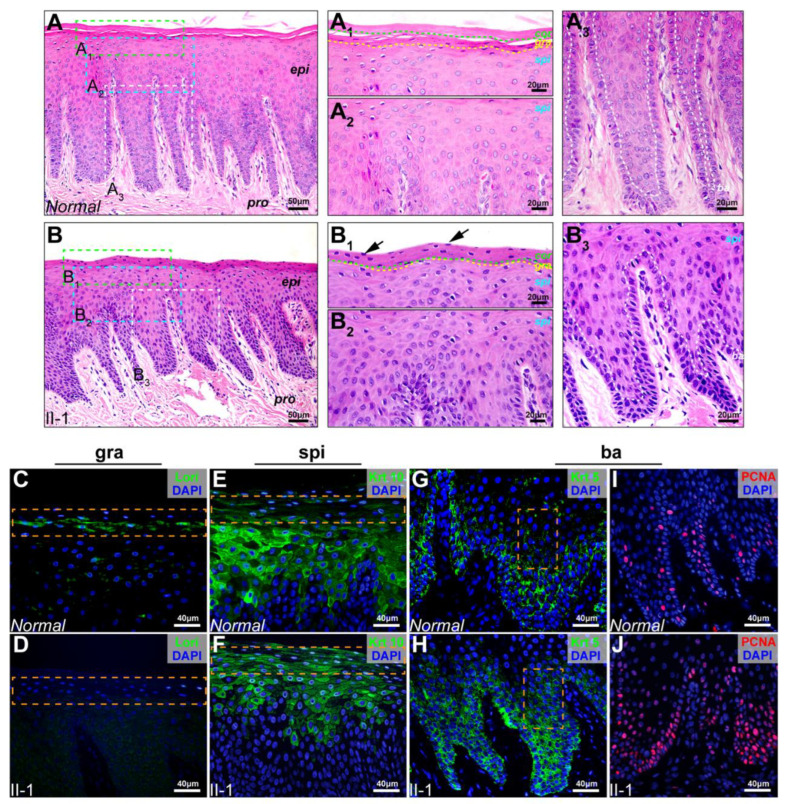

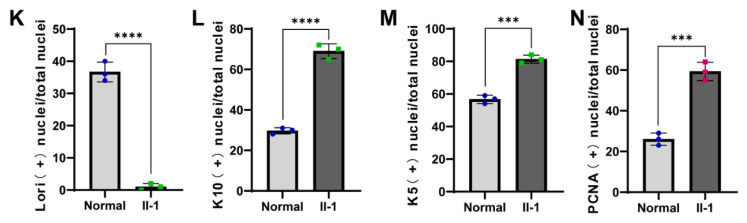
Histopathological assessment of the keratinized gingiva from the *KDF1* variant proband identified in this study. (**A**) HE staining of keratinized gingival epithelium sections from a normal control. Scale bars: 50 μm. (**B**) HE staining of keratinized gingival epithelium sections from the #285 proband II-1. Scale bars: 50 μm. (**A_1_**–**A_3_**,**B_1_**–**B_3_**) Higher magnification fields of stratum corneum (**A_1_**,**B_1_**), stratum granulosum (**A_1_**,**B_1_**), stratum spinosum (**A_2_**,**B_2_**), and stratum basale (**A_3_**,**B_3_**). Green dotted lines denote the boundaries of the stratum corneum and stratum granulosum. Yellow dotted lines denote the boundaries of the stratum granulosum and stratum spinosum. White dotted lines denote the boundaries of the stratum spinosum and stratum basale. Scale bars: 20 μm. (**C**–**H**) Keratinized gingival epithelium sections from a normal control (**C**,**E**,**G**) and the #285 proband II-1 (**D**,**F**,**H**) were immunostained with different epithelial differentiation markers as indicated (Lor: loricrin, Krt10: keratin 10, Krt5: keratin 5). (**I**,**J**) Keratinized gingival epithelium sections from a normal control (**I**) and the #285 proband II-1 (**J**) were immunostained with proliferation marker PCNA. Scale bars: 40 μm. Orange dotted squares denote the zones of interest that were measured for quantitative statistics. (**K**–**N**) Quantitation of loricrin, keratin 10, keratin 5, and PCNA in corresponding interest zones (*n* = 3, two-tailed unpaired Student’s *t*-test). Error bar indicates mean ± SD, **** *p* < 0.0001; *** *p* < 0.001. Experiments were repeated at least three times. Loricrin-Normal vs. loricrin-II-1: *p* < 0.0001; keratin 10-Normal vs. keratin 10-II-1: *p* < 0.0001; keratin 5-Normal vs. keratin 5-II-1: *p* = 0.0003; PCNA-Normal vs. PCNA-II-1: *p* = 0.0004. epi: epithelium, pro: lamina propria, cor: stratum corneum, gra: stratum granulosum, spi: stratum spinosum, and ba: stratum basale.

Hematoxylin and eosin (HE) staining showed a disorganized oral epithelium structure in the proband with KDF1 variant R307P, and it was manifested as epithelial dyskeratosis, with a large number of residual nuclei in the keratinization layer (Figure 4A,B,A_1_,B_1_), indistinct stratum granulosum (Figure 4A,B,A_1_,B_1_), and densely stained cytoplasm of the stratum spinosum and stratum basal cells (Figure 4A,B,A_2_,B_2_,A_3_,B_3_). Immunofluorescence staining further demonstrated the ectopic expression of epithelial cell differentiation markers and increased basal cell proliferation in keratinized gingiva samples from the proband, including the loss of the stratum granulosum cell marker loricrin, which represented the late differentiation of the epithelium (Figure 4C,D), expansion of stratum spinosum cell marker keratin 10 and basal cell marker keratin 5 (Figure 4E–H), and significant hyperproliferation of the basal cells (Figure 4I,J). Therefore, these results demonstrated that KDF1 variant R307P caused proliferation and differentiation defects in the gingival epithelium, and they suggest that *KDF1* is essential for gingival epithelium stratification and development.

### 2.5. KDF1 Variant R307P Suppressed Wnt Signaling Activation

To confirm the pathogenicity of the novel KDF1 variant R307P identified in our study, we performed in vitro functional experiments. Previously reported KDF1 variants, namely, R303P, I275L, and F251L were also included for comparison. We constructed variant 293T cell models by overexpressing the wild type or KDF1 variants in 293T cells.

**Figure 5:**
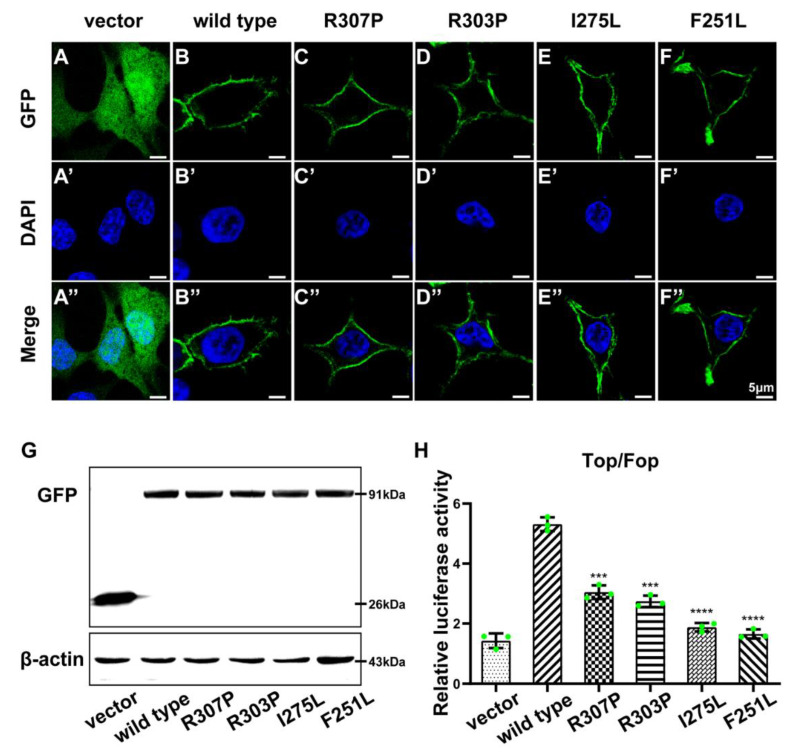
In vitro functional assessments of the KDF1 variant R307P identified in this study and the reported KDF1 variants in previous studies. (**A**–**F**) Fluorescence microscopy images illustrating the subcellular localization of the wild type (**B**) and four KDF1 variants reported to date: R307P (**C**), R303P (**D**), I275L (**E**), and F251L (**F**). An empty vector was used for transfection into 293T cells as a negative control (**A**). (**A’**–**F’**) Nuclei staining by DAPI. (**A”**–**F”**) Merge vision of GFP and DAPI. Scale bars: 5 μm. (**G**) Western blotting detects the expression of wild type and KDF1 variants at the protein level. (**H**) TOP-/FOP-flash luciferase assay assessing the activation of Wnt signaling in the wild type and KDF1 variants group (*n* = 3, two-tailed unpaired Student’s *t*-test). The error bar indicates mean ± SD, **** *p* < 0.0001; *** *p* < 0.001. Wild type vs. R307P: *p* = 0.0003; wild type vs. R303P: *p* = 0.0001; wild type vs. I275L: *p* < 0.0001; wild type vs. F251L: *p* < 0.0001. Experiments were repeated at least three times.

Fluorescence microscopy revealed that KDF1 was specifically expressed on the membrane of wild-type transfected cells, while extensive green fluorescence expression was observed in the negative control groups (Figure 5A–A”,B–B”). All four KDF1 variants were located in the same position as the wild type (Figure 5C–C”,F–F”), suggesting that the membrane localization of KDF1 was not affected. Western blotting confirmed the successful expression of GFP fusion proteins at the predicted molecular weight in the wild type and KDF1 variants (Figure 5G).

A recent report indicated that KDF1 activates the Wnt/β-catenin pathway, an important regulator of tooth and epithelial-mesenchymal interactions [25]. The impact of KDF1 variants on Wnt signaling activity was evaluated using the TOP-flash/FOP-flash luciferase reporter system. The results showed that the TOP-flash/FOP-flash transactivation activity was significantly reduced in the R307P-, R303P-, I275L-, and F251L-transfected groups (compared to the wild type, *p* < 0.001; Figure 5H), suggesting that Wnt signaling activation was suppressed by the KDF1 variants. These results also suggested that Wnt signaling may be involved in the pathogenic mechanism of KDF1-related anomalies.

## 3. Discussion

Our study identified a novel heterozygous *KDF1* variation (c.920G>C;p.R307P) in an autosomal-dominant inherited non-syndromic tooth agenesis family. This was the first study to report clinical features related to *KDF1*, particularly dental and oral epithelium morphological features. Such features include shovel-shaped incisors, *cornicione*-shaped premolars and molars, and oral epithelial defects. Therefore, only three *KDF1* variations have been reported to be associated with ectodermal dysplasia or non-syndromic tooth agenesis [22,23,26], and our 0.66% variation detection rate further substantiated the rarity of *KDF1* variations that contribute to tooth agenesis. Information about the molecular role of *KDF1* and the association of KDF1 variations with human developmental anomalies is quite limited. For the first time, we investigated the genetic relationship between *KDF1* variations and shovel-shaped incisors and *cornicione*-shaped premolars and molars, and we demonstrated that *KDF1* may play a crucial role in controlling tooth morphological development. Results from the HE and immunofluorescence staining demonstrated that *KDF1* variant R307P could cause striking abnormalities of the keratinized gingival epithelium in cell proliferation and differentiation, and suggested that *KDF1* is essential for the gingival epithelium development and the oral epithelial stratification. However, a fine dissection of the biological functions of *KDF1* during dental and oral epithelial morphogenesis using conditional knockout mouse models is urgently required.

Human KDF1 amino acid shares 90% identity with mouse Kdf1 and is highly conserved among mammals. Our results showed the 307th arginine was multi-species conserved, which implies the consequent amino acid change p.R307P may have significant impacts on the biological function of KDF1. Bioinformatic prediction using common online in silico variant prediction tools also demonstrated the deleterious effects of KDF1:p.R307P and confirmed its pathogenicity. Nonetheless, considering clinical cases on tooth agenesis caused by KDF1 variations are quite limited as yet, additional functional studies, including in vitro cell transfection with the variant plasmids, multi-omics analyses, or RNA-sequencing of the attainable tissue from the patients are required to authentically verify the biological consequences and the pathogenicity of KDF1 variants.

DUF4656 is a member of a domain of unknown function (DUF) family [27,28]. Variant R307P was identified in this study, and variants F251L, I275L, and R303P were previously reported [22,23,26]. All variants are located at the conserved DUF4656 (aa 48-398), revealing that DUF4656 is a hotspot region for germline variations that can be preferentially screened for genetic analysis of tooth agenesis patients. The conformational consequences according to the algorithmic prediction indicated that the R307P variation identified here represents a substitution of the nonpolar amino acid proline for the polar amino acid arginine at DUF4656, suggesting that polar amino acids may be important for the structural stability of DUF4656 and normal physiological function. Our results further imply that DUF4656 has a vital functional role in the KDF1 protein and mainly participates in the development of epithelial-derived tissues, such as the tooth and oral epithelium. However, the crystallographic structural analysis of KDF1 and the specific molecular functions of DUF4656 and its interacting proteins need to be further investigated.

Multipronged studies on tooth development using mouse models have shown that the reciprocal interactions of oral epithelial-mesenchymal tissues and the early epithelial signaling centers in the placode play an indispensable role in tooth morphogenesis [29,30,31]. However, the genetic mechanisms of *KDF1* variations in tooth agenesis cases are still unclear. The early epithelial signaling centers are mediated by four popular signaling pathways, Wnt, BMP, FGF, and Shh, and these pathways are particularly significant for primary enamel knot budding, which further determines tooth crown morphogenesis and late enamel formation [29,32,33,34,35,36,37]. A recent study demonstrated that KDF1 can activate the Wnt signaling pathway to regulate the epithelial-to-mesenchymal transition process in ovarian cancer [25]. Our results from Wnt luciferase assessments demonstrated that the novel KDF1 R307P variant and the other three reported variants suppressed Wnt signaling activation, suggesting that the loss-of-function of the variant allele might be the pathogenic mechanism of *KDF1* variation in tooth agenesis.

## 4. Methods and Materials

### 4.1. Recruitment of Studied Individuals

Healthy controls (*n* = 100) and a cohort of 151 probands, including 130 cases of non-syndromic tooth agenesis and 21 cases of ectodermal dysplasia, were recruited from the Department of Prosthodontics at the Peking University School and Hospital of Stomatology (PKUSS), Beijing, China. Other ectodermal organs, such as hair, skin, sweat glands, and nails, were also carefully inspected. Written informed consent was obtained from all participants for the use of blood or saliva samples and clinical data, and the publication of their photographs.

### 4.2. WES, Sanger Sequencing, and Segregation Analysis

Genomic DNA was isolated from peripheral blood using a Blood Genomic DNA Mini Kit (ComWin Biotech, Beijing, China), or from saliva using a GeneFix^TM^ saliva DNA isolation kit (Isohelix, Cell Projects, Kent, UK). The ANGEN Gene Medicine Technology Company (ANGEN, Beijing, China) was further commissioned for the WES analysis. In brief, orodental-related genes were annotated to preliminarily filter the detected gene variants [38], and then the remaining variants were selected by a minor allele frequency (MAF) ≤ 0.01 in the gnomAD database, and by the predicted functional impact through in silico bioinformatics tools (i.e., SIFT, PolyPhen-2, PROVEAN, and MutationTaster). The pathogenicity scores of the identified variants were determined according to the ACMG variant classification guidelines [39].

Familial segregation analysis was conducted via Sanger sequencing of attainable nuclear family members to verify candidate variations. PCR primers for *KDF1* coding exons for Sanger sequencing were designed using Primer-BLAST tools. The primer sequences are available upon request.

### 4.3. Conservation and Structural Prediction of the KDF1 Protein

For the conservation analysis, the KDF1 amino acid sequences of multiple species were obtained from the National Center for Biotechnology Information (NCBI), and an alignment analysis of the affected amino acids was conducted using ClustalX 2.1.

The effects of the KDF1 variant on the secondary protein structure were predicted using PsiPred 4.0. The referenced tertiary protein structure of wild-type KDF1 was predicted using the AlphaFold Protein Structure Database (UniProt Accession: Q8NAX2), and the conformational structural changes caused by the KDF1 R307P variant were analyzed using the PyMOL Molecular Graphics System (DeLano Scientific, San Francisco, CA, USA).

### 4.4. Intraoral Scanning and Cone-Beam Computed Tomography (CBCT)

The diagnosis of permanent tooth agenesis was confirmed using panoramic radiography. To further examine the morphological characteristics of the remaining teeth, digital intraoral scanning (3Shape Trios Standard-P11, Copenhagen, Denmark) and CBCT (VGi evo, NewTom, Verona, Italy) were performed. The voxel resolution of the CBCT was 250 μm.

### 4.5. Assessment of Dental Enamel Structures

To assess the characteristics of the enamel structure, we performed SEM and ground sections of the lower right lateral deciduous incisors extracted from #285 proband with *KDF1* variation and a normal control. All teeth were fixed in 10% neutral buffered formalin and rinsed with an ultrasonic cleaner. For the SEM analyses, the teeth were air-dried, split along the buccolingual direction, mounted on aluminum stubs, and sputter-coated with gold. The enamel structures of the deciduous teeth of patients with *KDF1* variation were visualized using a scanning electron microscope (HITACHI S4800; HITACHI, Tokyo, Japan).

For ground sections, the teeth were embedded in methyl methacrylate, sectioned at a thickness of 100 μm using a Leitz 1600 (Leica Biosystem, Nußloch, Germany) along the longitudinal axis of the tooth, dehydrated in an ethanol series, cleared with xylene, and mounted in neutral gum. Unstained ground sections of undemineralized teeth were observed under a microscope.

### 4.6. Histomorphological Analysis of the Oral Epithelium

For HE staining, the samples were processed and stained according to standard procedures. The keratinized gingival epithelium was obtained from the proband with the *KDF1* variation and compared with the epithelium from normal controls. 

Immunostaining was performed according to the standard procedures [40]. Primary antibodies against loricrin (Proteintech-55439-1-AP, 1/50, Chicago, IL, USA), keratin 5 (Biolegend-905503, 1/50, San Diego, CA, USA), keratin 10 (Biolegend-905403, 1/50), and PCNA (Proteintech-10205-2-AP, 1/100) were used. Alexa Fluor 488 and 594 (ZSGB-BIO-ZF-0516, Beijing, China) were used for detection. Slides were mounted using a mounting medium with DAPI (ZSGB-BIO-ZLI-9557), and images were captured using a fluorescence microscope. Evaluation of epithelial differentiation and proliferation markers was performed using ImageJ software 1.8.0 (NIH, Bethesda, MD, USA). 

### 4.7. Construction of Plasmids

The full-length coding region of the human *KDF1* gene was subcloned into the pEGFP-N1 expression vector between 5′-NheI and 3′-BamHI to construct the *KDF1* wild-type plasmid. In vitro, site-directed mutagenesis was performed to generate the newly identified R307P variant plasmid and three previously reported variant plasmids (F251L, I275L, and R303P). All plasmids were synthesized and verified by Tsingke Biotechnology Co. Ltd. (Tsingke, Beijing, China).

### 4.8. Cell Culture, Transient Transfection, and Western Blotting

Human embryonic kidney 293T (HEK-293T) cells were cultured in Dulbecco’s modified Eagle’s medium (Invitrogen, Grand Island, NY, USA) supplemented with 10% fetal bovine serum (Analysis Quiz, Beijing, China) and 1% penicillin-streptomycin (Beyotime, Shanghai, China) at 37 °C in the presence of 5% CO_2_ and 95% air. Transient transfections of wild-type and variant plasmids were performed using Lipofectamine™ 3000 Transfection Reagent (Invitrogen), according to the manufacturer’s instructions. Forty-eight hours after transfection, the transfected cells were lysed using RIPA buffer (Solarbio, Beijing, China), and protein quantification was performed using the bicinchoninic acid protein concentration assay (BCA) kit (Invitrogen). Western blotting was then performed using 40 μg of total protein in each group to examine the efficiency. Blots were probed with anti-GFP (Abcam-ab1218, 1/1000, Cambridge, UK) and anti-β-actin (Sungene-KM9006T, 1/1000, Tianjin, China) antibodies. Protein bands were visualized using an ECL reagent (NCM Biotech, Suzhou, China) and imaged.

### 4.9. Fluorescence Microscopy

293T cells transfected with the *KDF1* wild-type and four variant plasmids for forty-eight hours were fixed with 4% paraformaldehyde and mounted with an antifade mounting medium containing DAPI (ZSGB-BIO). The subcellular expression pattern was observed using a Leica TCS-SP8 confocal microscope (Leica, Berlin/Heidelberg, Germany) with a ×40/1.00 numerical aperture oil objective lens.

### 4.10. Luciferase Activity Detection

*KDF1* wild-type 293T cells and four variant 293T cells were co-transfected with either a TOP-flash or FOP-flash reporter plasmid. All groups were simultaneously transfected with *Renilla* reporter plasmids (*phRL-TK*; Promega, Madison, Wisconsin, USA) as endogenous controls. After cell transfection, protein lysates were extracted for the measurement of firefly and Renilla luciferase activities using a Dual-Luciferase Reporter kit (Promega) in triplicate. The firefly luciferase activity of each group was normalized to that of Renilla luciferase. Wnt/β-catenin activation was determined as the ratio of TOP-flash firefly activity to FOP-flash firefly activity. Experiments were repeated at least three times.

### 4.11. Quantitative Analysis 

Quantification of epithelial differentiation or proliferation markers was performed using GraphPad Prism software. Student’s *t*-test was used to evaluate statistically significant differences (*p* < 0.05). Data are presented as the mean ± SD (*n* = 3).

## 5. Conclusions

In this study, we identified the previously undescribed *KDF1* heterozygous missense variation c.920G>C;p.R307P in two individuals segregated from one non-syndromic tooth agenesis family. Our study describes unprecedented anomalies in tooth morphology and gingival epithelial structure caused by the pathogenic heterozygous *KDF1* missense variation c.920G>C;p.R307P. Our results broaden the *KDF1*-related genotype and phenotype spectrum, and they suggest a genetic association of *KDF1* with tooth and oral epithelial morphogenesis.

## Figures and Tables

**Table 1:** Pathogenic prediction of the novel *KDF1* variant.

Patients	Exon/ Domain	Nucleotide/ Protein Change	Variant	Mutation Taster	PROVEAN	SIFT	PolyPhen-2	gnomAD, dbSNP, 1000 G	ACMG Classification (Evidence of Pathogenicity)
#285 II-1 #285 I-1	2/DUF4656	c.920G>C/ p.R307P	missense	Disease causing	−6.41 Deleterious	0.000 Damaging	0.341 Benign	Not present	Likely pathogenic PM1 + PM2 + PP1 + PP3

## Data Availability

The variations identified in this study were submitted to the ClinVar database (submission ID SCV001984771). WES data are available from the SRA database (accession number PRJNA782853).
